# Different Levels of CEA, CA153 and CA125 in Milk and Benign and Malignant Nipple Discharge

**DOI:** 10.1371/journal.pone.0157639

**Published:** 2016-06-21

**Authors:** Song Zhao, Yu Mei, Jianli Wang, Kai Zhang, Rong Ma

**Affiliations:** 1 Department of Breast Surgery, Qilu Hospital of Shandong University, Jinan, Shandong, China; 2 Department of Breast Surgery, Jinan Maternity and Child Care Hospital, Jinan, Shandong, PR China; 3 Department of Pathophysiology, School of Medicine, Shandong University, Shandong, China; The Ohio State University, UNITED STATES

## Abstract

**Background:**

The aim of this study was to assess the diagnostic values of three breast tumor markers (i.e., CEA, CA153 and CA125) in milk and nipple discharge in the prediction of different breast diseases diagnoses.

**Methods:**

Three hundred thirty-six patients (96 breast cancer and 240 benign disease patients) with nipple discharge and a control group of 56 healthy parturient participants were enrolled in the present study. Nipple discharge samples were preoperatively collected from the patients, and milk was collected from the colostrum of the parturient participants. The samples were assayed for the CEA, CA153 and CA125 levels. Cutoff values were determined for the detection of breast diseases using ROC curves.

**Results:**

The levels of CEA, CA153 and CA125 were significantly different between the nipple discharge and the milk (all *ps <* 0.001). In the nipple discharge, the CEA and CA153 levels in the breast cancer group were significantly greater than those in the benign group (all *ps <* 0.001), and cutoff values of 263.3 ng/mL and 1235.3 U/mL, respectively, were established. However, the expression of CA125 did not differ significantly between the breast cancer and benign groups.

**Conclusion:**

Differences in the apparent expression levels of CEA, CA153 and CA125 in patients with nipple discharge and healthy persons were validated. The present data suggest that CEA and CA153 might potentially be useful in the differential diagnoses of benign tumors and breast cancer. CA125 did not seem to be useful for breast cancer detection.

## Introduction

In addition to breast masses and breast pain, nipple discharge is also a relatively common breast complaint that accounts for up to 5% of the reasons for which women seek medical advice [1, 2]. Patients with nipple discharge have pathological outcomes. The majority of nipple discharge are spontaneous. Discharge was located in or originate from the mammary ducts and are generally associated with benign or malignant breast tumors [3]. Among patients who present with nipple discharge, the incidence of malignancy has been reported to range from 5% to 21% [4–6]. A large portion of patients with nonpuerperal nipple discharge have symptoms of spontaneous unilateral serous or bloody discharge [7]. The evaluation options for nipple discharge include ultrasonography, exfoliative cytologic analysis, galactography and ductoscopy. However, imaging examinations are not of great clinical value in the differential diagnosis of breast cancer in patients without palpable masses. Galactography is considered a method for determining the locations and extents of abnormalities in the duct [5, 8, 9]. Mammography can miss 10 to 40% of early breast cancers [10–13].

Nipple discharge in the ducts of nonlactating women contain concentrated secreted proteins from the breast ductal epithelium [14–16]. Carcinoembryonic antigen (CEA) is useful for the diagnosis of recurrence and the prognosis of breast cancer [17, 18]. Cancer antigen 153 (CA153) is used in the management of the prognoses, metastases and recurrences of breast cancer patients [19–21]. The preoperative levels of CEA and CA153 in the serum are well known to significantly influence the prognosis of breast cancer [22, 23]. Cancer antigen 125 (CA125) has been found to be up-regulated in breast cancer tissues and not expressed in non-neoplastic ducts [24]. There is a need to identify an attractive option for the screening of nipple discharge samples that are simply and non-invasively obtained. The aim of this study was to determine the diagnostic values of the CEA, CA153 and CA125 levels in nipple discharge compared the milk of healthy controls. The present study also sought to compare tumor marker levels between the milk of normal lactating women and the nipple discharge of patients with breast cancer or benign lesions.

## Materials and Methods

### Patients

A group of 336 patients with complaints of nipple discharge were scheduled to undergo and a group of 56 puerperal healthy controls were included in our study, which was conducted at Qilu Hospital of Shandong University from February 2012 to May 2015. The study cohort included women with unilateral nipple discharge. The patients had received no preoperative treatment. The group of patients with nipple discharge was further divided into two groups (i.e., breast cancer and benign groups) according to postoperative pathological diagnoses. The healthy controls had no histories of cancer or breast diseases.

This study complied with the standards of the Declaration of Helsinki and the current ethical guidelines and was approved by the institutional ethical committee of Qilu Hospital of Shandong University. Written informed consent for participation in the study was obtained from all of the patients.

### Samples

All samples were collected before any treatment was initiated and within 2 days of hospitalization. The nipple was first cleansed with alcohol swabs to remove cellular debris. The nipple discharge and milk were expressed via manual compression of the breast. No complications occurred. Droplets of nipple discharge or milk were collected in an Eppendorf tube. The tube was then stored in a dedicated refrigerator at 4°C. The quantities of the collected nipple discharge varied from 20 μL to 200 μL. The samples were transported to the laboratory department within 8 hours of collection. Viscous samples were diluted up to 20-fold with normal saline prior to centrifugation and storage at 4°C. The concentrations of CEA, CA153 and CA125 in the nipple discharge and milk samples were quantitatively measured via an automated test system that utilized sandwich electro-chemiluminescence immunoassay (ECLIA) kits (Roche cobas e601 analyzer, Roche Diagnostics, Indianapolis, USA). All tumor marker assays were performed at Qilu Hospital of Shandong University according to manufacturer’s protocol. The laboratory personnel were blinded to the clinical information. Commercial reference control sera were used for quality control and calibration.

### Statistics

The patients’ characteristics were descriptively summarized. The median and interquartile ranges were used to express the distributions of the tumor markers in the nipple discharge and milk. Comparisons of the data between the groups were performed using Mann-Whitney U tests. Receiver operating characteristic (ROC) analyses were used to compare the diagnostic performances of the different tumor markers. Risk scores were assigned to all participants according to a linear combination of the expression levels of the tumor markers that were weighted according to the regression coefficients. Cut-off values were determined as the parameter value that maximized the sum of the specificity and sensitivity. Positive predictive values (PPVs), negative predictive values (NPVs) and the areas under the receiver operating characteristic curves were calculated based on the cutoff values for CEA and CA153. All *p* values were two-sided, and *p*<0.05 was considered statistically significant. The statistical analyses were performed using SPSS 17.0 software (SPSS Inc., Chicago, USA).

## Results

### Characteristics of the study populations

The characteristics of participants were summarized. This patient group included 96 patients (median age 49, 25–76) with breast cancer and 240 patients (median age 43, 17–79) with benign lesions. The control group (n = 56) included puerperal lactating women (median age 31, 19–42). Ninety-six patients were postoperatively pathologically confirmed to have breast cancer including the following histological subtypes: invasive ductal carcinoma (n = 35), ductal carcinoma in situ (n = 32), and intraductal papillary carcinoma (n = 29). Two hundred forty patients were diagnosed with benign breast diseases that included intraductal papilloma (n = 182) and mammary duct ectasia (n = 58).

### Tumor marker levels

To study of the relationships of the CEA, CA153 and CA125 levels with breast disease, the tumor markers levels from the cancerous breasts, the breasts with benign diseases, and the breasts of the healthy controls were compared. The levels of CEA, CA153 and CA125 were significantly higher in the samples from the breast cancer patients than in the control and benign samples (all *ps<*0.001; Table 1). The median levels of CEA, CA153 and CA125 were elevated by 38.7-, 3.9- and 7-fold, respectively, in the patients with breast cancer compared with the healthy lactating controls. All of the differences between the breast cancer group and healthy controls were significant (all *p<*0.001). The median tumor marker expression level exhibited a trend toward higher values in the patients with benign lesions compared with the controls. The levels of CEA and CA125 in the nipple discharge from the patients with benign lesions were significantly higher than those of the healthy controls with the exception of CA153 (*p<*0.001 for CEA and CA125, and *p* = 0.268 for CA153). The differences in the CEA and CA153 expressions between the discharge from the breast cancer patients and those with benign lesions were significant (all *ps <* 0.001). No significant difference in CA125 was observed between the breasts with breast cancer and those with benign disease (*p* = 0.493).

**Table 1:** Tumor markers in milk and nipple discharge*.

Tumor Markers	Breast cancer group (n = 96)	Benign group (n = 240)	Milk (n = 56)	*p* Value^#^
CEA, ng/mL	375.4 (137.4–1662.8)^§^	73.0 (26.4–173.1)	9.7 (4.6–27.8)^◎^	*p<*0.001
CA153, U/mL	1405.2 (466.0–3479.4)^§^	435.4 (121.3–1291.7)	363.1 (178.1–627.6)	*p<*0.001
CA125, U/mL	15988.0 (4986.0–48860.0)	21220.0 (4872.0–69780.0)	2296.0 (972.5–5428.0)^◎^	*p<*0.001

*Data are presented as median (quartiles).

^#^Significance level of Kruskal-Wallis test.

^§^Significantly higher than the respective values in benign and healthy control groups.

^◎^Significantly lower than the respective values in breast cancer and benign groups.

### Diagnostic performances of CEA, CA153 and CA125 in the differentiation of breast cancer and benign disease

**Fig 1:**
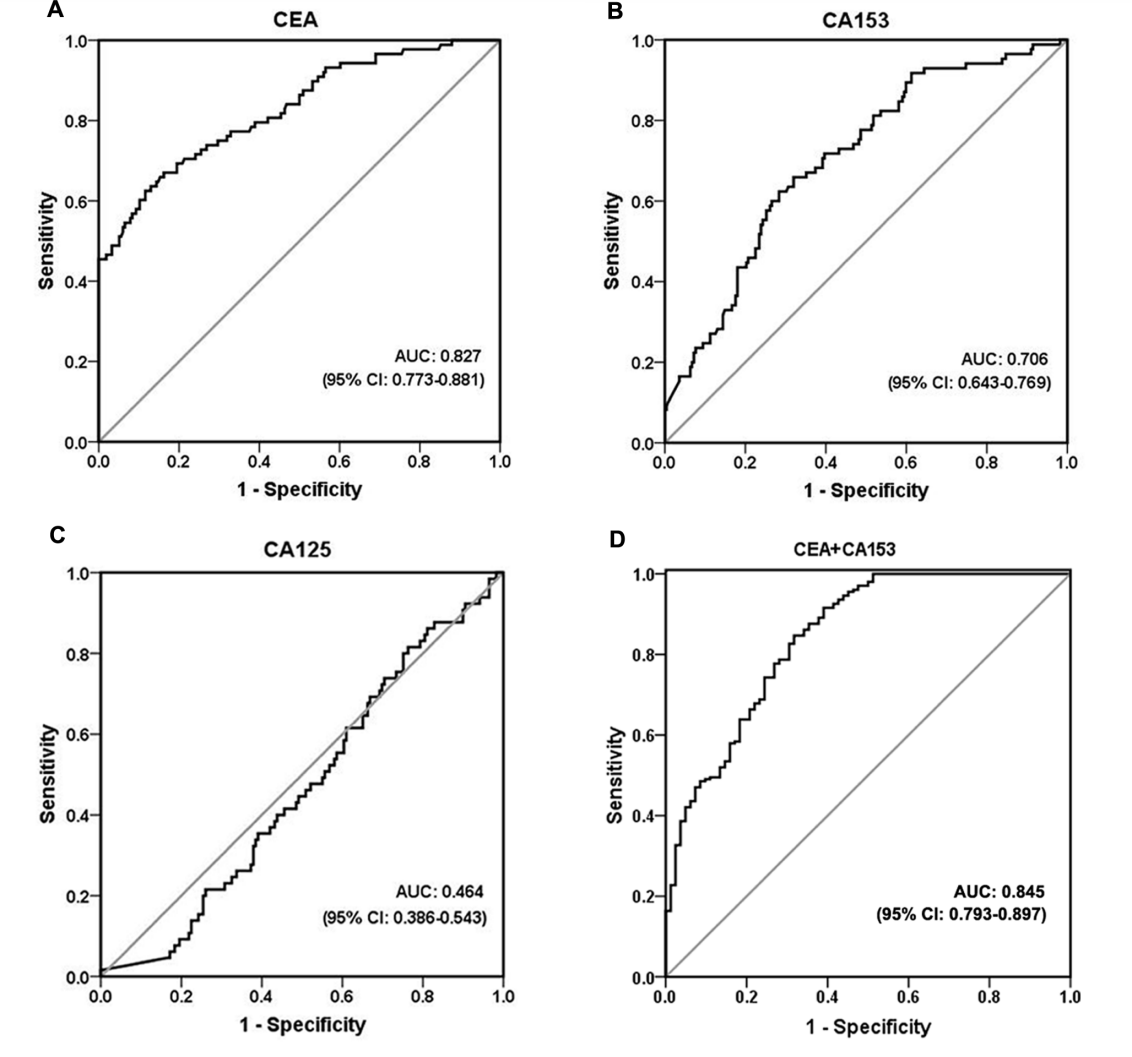
Receiver operating characteristic (ROC) curve analyses. The ROC plots for CEA (A), CA153 (B), CA125 (C) and the combination of CEA and CA153 (D) were used to differentiate breast cancer from benign disease. AUC, area under the receiver operating characteristic curve; CI, confidence interval.

Fig 1 illustrates the ROC curves for each marker and the corresponding areas under the ROC curves (AUCs). ROC analyses indicated that CEA was the best performing marker in terms of the differentiation of breast cancer from benign disease (*p<*0.001). The AUCs were 0.827 for CEA, 0.706 for CA153, and 0.464 for CA125. According to a non-parametric Mann-Whitney U test, the CA125 levels were not significantly different (*p* = 0.397) between the breast cancer and benign groups. The predicted probabilities of breast cancer diagnoses based on the CEA and CA153 levels were used to construct a ROC curve. The AUC for the combined tumor markers was 0.845 (95% confidence interval [CI], 0.793 to 0.897), and the sensitivity and specificity were 84.0% and 74.5%, respectively (Fig 1D). The cutoff values for CEA and CA153 (and the combination of these markers) with *ps<*0.05 were determined based on the maximum sums of the sensitivities and specificities are presented in Table 2. We used cutoff points of 263.3 ng/mL for CEA and 1235.3 U/mL for CA153, which yielded specificities of 88.4% and 71.6%, respectively, and sensitivities of 62.5% and 62.4%, respectively. Table 2 displays the sensitivities, specificities, PPVs and NPVs for both tumor markers. CEA exhibited the highest specificity (88.4%), although its sensitivity was relatively low (62.5%), and CA153 exhibited a lower specificity (71.6%). CEA and CA153 exhibited similar NPVs (85.3% and 82.2%, respectively), and relatively low PPVs were noted (69.6% and 46.7%, respectively). The combination of CEA with CA153 resulted in some non-significant changes in the abovementioned parameters. The combination exhibited a significantly greater sensitivity (84.0%) than CEA or CA153 alone.

**Table 2:** Sensitivity, specificity, and areas under the curves for CEA, CA153 and combinations of these markers in nipple discharge with breast cancer.

Tumor markers	Cut-off values	Sensitivity (%)	Specificity (%)	PPV (%)	NPV (%)	AUC (95% CI)	*p* values
CEA, ng/mL	263.3	62.5	88.4	69.6	85.3	0.827 (0.773–0.881)	*p<*0.001
CA153, U/mL	1235.3	62.4	71.6	46.7	82.2	0.706 (0.643–0.769)	*p<*0.001
CEA and CA153	0.799	84	74.5	56.1	87.6	0.845 (0.793–0.897)	*p<*0.001

CEA, carcinoembryonic antigen; CA153, cancer antigen 153; AUC, area under the curve; CI, confidence interval.

## Discussion

In agreement with previous studies [25–27], the most common cause of nipple discharge was intraductal papilloma, and nipple discharge was the presenting symptom for cancer in the present study. Our data revealed a papilloma rate of 54.2% (182 of 336) and cancer rate of 28.6% (96 of 336) among the patients with nipple discharge.

In a previous paper, the serum CEA, CA153 and CA125 levels were demonstrated to be of great value in clinical diagnoses and to provide details for the management of breast cancer recurrence and metastasis. The associations of high levels of serum CEA and CA153 with poor prognoses have been validated [28, 29]. In patients with early or localized breast cancer, the serum CA153 levels do not clinically benefit diagnosis [30]. There are some limitations of the use of serum tumor markers in the diagnosis of breast cancer, such as relatively limited sensitivity and specificity [20, 31]. In the present study, we selected three tumor markers (i.e., CEA, CA153 and CA125) that are related to breast cancer and examined their expression levels in nipple discharge samples obtained from patients with benign and malignant breast diseases and milk samples from healthy puerperal women. The marker levels were higher in the nipple discharge than in the serum and exhibited greater diagnostic specificity probably because they are produced locally and released directly into the mammary ducts.

There is a paucity of reports that have examined the diagnostic values of the three abovementioned tumor markers for breast lesions with nipple discharge [16, 32, 33]. A literature search revealed that there are no references for the levels of these markers in milk. We demonstrated that the levels of CEA, CA153 and CA125 were higher in the patients with breast lesions than in the healthy controls. These findings suggest that all three markers were indeed different between the pathological nipple discharge and normal milk samples. The present results also revealed that the expression levels of all three markers in the cancer group were significantly greater than those in the healthy control. Elevated tumor marker levels occur during the process of the development of tumors from nonexistent to malignant. CEA and CA125 were expressed at different levels in the benign group and the healthy controls. No significant difference in the CA153 levels between the benign breast disease patients and the healthy controls was observed. We conclude that qualitative changes occurred during malignant transformation and may have resulted in an increase in the level of CA153 in the nipple discharge. In the present study, only the elevation in the CEA level was associated with the progression from a normal state to benign disease to malignant disease.

ROC curves were utilized to compare the tumor marker levels between the patients with benign and malignant diseases. CEA exhibited a greater AUC compared with the other tumor markers and thus better differentiated between the patients with breast cancer and those with benign diseases (0.827 vs. 0.706 and 0.464). CA125 did not significantly differ between the breast cancer and benign groups. As a potential marker in nipple discharge, CA125 has exhibited limited reliability in the differentiation of benign and malignant breast lesions. The diagnostic values of the CEA and CA153 levels in the nipple discharge were 263.3 ng/mL and 1235.3 U/mL, respectively, and these markers exhibited relatively satisfactory specificities (88.4% and 71.6%, respectively). Additionally, the diagnostic performance of the combination of the two markers (CEA and CA153) was comparable to those of the individual markers. ROC analysis revealed that the AUC for the combination of markers was at the cutoff of 0.845. Moreover, the sensitivity increased to 84.0%. However, the combined markers exhibited good sensitivity but relatively poor specificity in the diagnosis of breast cancer.

Summarizing our results, we recommend that CEA and CA153 should be used as informative tumor markers in the diagnosis of breast cancer with nipple discharge. We suggest that the measured cutoff values for CEA and CA153 be used for patients with nipple discharge because these values were able to differentiate between cancer and benign lesions. The determination of tumor markers in nipple discharge has been proposed as an alternative, noninvasive method of establishing the diagnosis of breast cancer. We recommend that patients with elevated CEA and CA153 levels suggestive of breast cancer receive subsequent examinations or clinical interventions.

We are aware of some potential limitations of the present study. The samples included in the study came only from Qilu Hospital of Shandong University, which might have influenced the representativeness of the research group. Further research involving multi-center studies is necessary to achieve a better understanding of the relationship between tumor markers and breast diseases. We did not evaluate whether high CEA, CA153 and CA125 tumor marker levels reflected high tumor burden or poor outcomes. Studies with long-term follow-up data regarding patient prognoses and survival rates are necessary.

In conclusion, the use of the expression levels of CEA, CA153 and CA125 to distinguish patients with nipple discharge from healthy people were validated. CEA and CA153 levels in nipple discharge were useful for differentiating between breast cancer patients and those with benign tumors. CA125 did not seem to be useful for breast cancer detection. The combination of CEA and CA153 exhibited considerable clinical value for the diagnosis of breast cancer and a much greater sensitivity than CEA or CA153 alone. We propose that CEA and CA153 could be used to develop a non-invasive screening tool for the diagnostic evaluation of nipple discharge.

## References

[1] Vargas HI, Romero L, Chlebowski RT. Management of bloody nipple discharge. Current treatment options in oncology. 2002;3(2):157–61. . 12057078 10.1007/s11864-002-0061-9

[2] Baitchev G, Gortchev G, Todorova A, Dikov D, Stancheva N, Daskalova I. Intraductal aspiration cytology and galactography for nipple discharge. International surgery. 2003;88(2):83–6. . 12872900

[3] Sauter ER, Klein-Szanto A, Macgibbon B, Ehya H. Nipple aspirate fluid and ductoscopy to detect breast cancer. Diagnostic cytopathology. 2010;38(4):244–51. 10.1002/dc.21177 19795490 PMC3390775

[4] Louie LD, Crowe JP, Dawson AE, Lee KB, Baynes DL, Dowdy T, et al Identification of breast cancer in patients with pathologic nipple discharge: does ductoscopy predict malignancy? American journal of surgery. 2006;192(4):530–3. 10.1016/j.amjsurg.2006.06.004 . 16978968

[5] Montroni I, Santini D, Zucchini G, Fiacchi M, Zanotti S, Ugolini G, et al Nipple discharge: is its significance as a risk factor for breast cancer fully understood? Observational study including 915 consecutive patients who underwent selective duct excision. Breast cancer research and treatment. 2010;123(3):895–900. 10.1007/s10549-010-0815-1 . 20354781

[6] Simpson JS, Connolly EM, Leong WL, Escallon J, McCready D, Reedijk M, et al Mammary ductoscopy in the evaluation and treatment of pathologic nipple discharge: a Canadian experience. Canadian journal of surgery Journal canadien de chirurgie. 2009;52(6):E245–8. 20011159 PMC2792391

[7] Furuya C, Kawano H, Yamanouchi T, Oga A, Ueda J, Takahashi M. Combined evaluation of CK5/6, ER, p63, and MUC3 for distinguishing breast intraductal papilloma from ductal carcinoma in situ. Pathology international. 2012;62(6):381–90. 10.1111/j.1440-1827.2012.02811.x . 22612506

[8] Ohlinger R, Stomps A, Paepke S, Blohmer JU, Grunwald S, Hahndorf W, et al Ductoscopic detection of intraductal lesions in cases of pathologic nipple discharge in comparison with standard diagnostics: the German multicenter study. Oncology research and treatment. 2014;37(11):628–32. 10.1159/000368338 . 25427580

[9] Kamali S, Bender O, Kamali GH, Aydin MT, Karatepe O, Yuney E. Diagnostic and therapeutic value of ductoscopy in nipple discharge and intraductal proliferations compared with standard methods. Breast cancer. 2014;21(2):154–61. 10.1007/s12282-012-0377-7 . 22669683

[10] Tice JA, Miike R, Adduci K, Petrakis NL, King E, Wrensch MR. Nipple aspirate fluid cytology and the Gail model for breast cancer risk assessment in a screening population. Cancer epidemiology, biomarkers & prevention: a publication of the American Association for Cancer Research, cosponsored by the American Society of Preventive Oncology. 2005;14(2):324–8. 10.1158/1055-9965.EPI-04-0289 . 15734953

[11] Kolb TM, Lichy J, Newhouse JH. Comparison of the performance of screening mammography, physical examination, and breast US and evaluation of factors that influence them: an analysis of 27,825 patient evaluations. Radiology. 2002;225(1):165–75. 10.1148/radiol.2251011667 . 12355001

[12] Taylor K, Ames V, Wallis M. The diagnostic value of clinical examination and imaging used as part of an age-related protocol when diagnosing male breast disease: an audit of 1141 cases from a single centre. Breast. 2013;22(3):268–72. 10.1016/j.breast.2013.03.004 . 23570843

[13] Moon HJ, Jung I, Park SJ, Kim MJ, Youk JH, Kim EK. Comparison of Cancer Yields and Diagnostic Performance of Screening Mammography vs. Supplemental Screening Ultrasound in 4394 Women with Average Risk for Breast Cancer. Ultraschall in der Medizin. 2015;36(3):255–63. 10.1055/s-0034-1366288 . 24764212

[14] Deutscher SL, Dickerson M, Gui G, Newton J, Holm JE, Vogeltanz-Holm N, et al Carbohydrate antigens in nipple aspirate fluid predict the presence of atypia and cancer in women requiring diagnostic breast biopsy. BMC cancer. 2010;10:519 10.1186/1471-2407-10-519 20920311 PMC2958935

[15] Olopade OI, Pichert G. Cancer genetics in oncology practice. Annals of oncology: official journal of the European Society for Medical Oncology / ESMO. 2001;12(7):895–908. . 11521793 10.1023/a:1011176107455

[16] Sauter ER, Wagner-Mann C, Ehya H, Klein-Szanto A. Biologic markers of breast cancer in nipple aspirate fluid and nipple discharge are associated with clinical findings. Cancer detection and prevention. 2007;31(1):50–8. 10.1016/j.cdp.2006.12.004 17317033 PMC1865519

[17] Stieber P, Nagel D, Blankenburg I, Heinemann V, Untch M, Bauerfeind I, et al Diagnostic efficacy of CA 15–3 and CEA in the early detection of metastatic breast cancer-A retrospective analysis of kinetics on 743 breast cancer patients. Clinica chimica acta; international journal of clinical chemistry. 2015;448:228–31. 10.1016/j.cca.2015.06.022 . 26160053

[18] Shao Y, Sun X, He Y, Liu C, Liu H. Elevated Levels of Serum Tumor Markers CEA and CA15-3 Are Prognostic Parameters for Different Molecular Subtypes of Breast Cancer. PloS one. 2015;10(7):e0133830 10.1371/journal.pone.0133830 26207909 PMC4514648

[19] Sturgeon CM, Duffy MJ, Stenman UH, Lilja H, Brunner N, Chan DW, et al National Academy of Clinical Biochemistry laboratory medicine practice guidelines for use of tumor markers in testicular, prostate, colorectal, breast, and ovarian cancers. Clinical chemistry. 2008;54(12):e11–79. 10.1373/clinchem.2008.105601 . 19042984

[20] Duffy MJ, Evoy D, McDermott EW. CA 15–3: uses and limitation as a biomarker for breast cancer. Clinica chimica acta; international journal of clinical chemistry. 2010;411(23–24):1869–74. 10.1016/j.cca.2010.08.039 . 20816948

[21] Kim MJ, Park BW, Lim JB, Kim HS, Kwak JY, Kim SJ, et al Axillary lymph node metastasis: CA-15-3 and carcinoembryonic antigen concentrations in fine-needle aspirates for preoperative diagnosis in patients with breast cancer. Radiology. 2010;254(3):691–7. 10.1148/radiol.09091031 . 20123899

[22] Wu SG, He ZY, Zhou J, Sun JY, Li FY, Lin Q, et al Serum levels of CEA and CA15-3 in different molecular subtypes and prognostic value in Chinese breast cancer. Breast. 2014;23(1):88–93. 10.1016/j.breast.2013.11.003 . 24291374

[23] Lee JS, Park S, Park JM, Cho JH, Kim SI, Park BW. Elevated levels of preoperative CA 15–3 and CEA serum levels have independently poor prognostic significance in breast cancer. Annals of oncology: official journal of the European Society for Medical Oncology / ESMO. 2013;24(5):1225–31. 10.1093/annonc/mds604 . 23230137

[24] Lakshmanan I, Ponnusamy MP, Das S, Chakraborty S, Haridas D, Mukhopadhyay P, et al MUC16 induced rapid G2/M transition via interactions with JAK2 for increased proliferation and anti-apoptosis in breast cancer cells. Oncogene. 2012;31(7):805–17. 10.1038/onc.2011.297 21785467 PMC3288594

[25] Kapenhas-Valdes E, Feldman SM, Cohen JM, Boolbol SK. Mammary ductoscopy for evaluation of nipple discharge. Annals of surgical oncology. 2008;15(10):2720–7. 10.1245/s10434-008-0012-1 . 18685898

[26] Vaughan A, Crowe JP, Brainard J, Dawson A, Kim J, Dietz JR. Mammary ductoscopy and ductal washings for the evaluation of patients with pathologic nipple discharge. The breast journal. 2009;15(3):254–60. 10.1111/j.1524-4741.2009.00714.x . 19645780

[27] Dooley WC. Routine operative breast endoscopy for bloody nipple discharge. Annals of surgical oncology. 2002;9(9):920–3. . 12417516 10.1007/BF02557531

[28] Harris L, Fritsche H, Mennel R, Norton L, Ravdin P, Taube S, et al American Society of Clinical Oncology 2007 update of recommendations for the use of tumor markers in breast cancer. Journal of clinical oncology: official journal of the American Society of Clinical Oncology. 2007;25(33):5287–312. 10.1200/JCO.2007.14.2364 . 17954709

[29] Bidard FC, Hajage D, Bachelot T, Delaloge S, Brain E, Campone M, et al Assessment of circulating tumor cells and serum markers for progression-free survival prediction in metastatic breast cancer: a prospective observational study. Breast cancer research: BCR. 2012;14(1):R29 10.1186/bcr3114 22330883 PMC3496147

[30] Stieber P, Molina R, Chan DW, Fritsche HA, Beyrau R, Bonfrer JM, et al Clinical evaluation of the Elecsys CA 15–3 test in breast cancer patients. Clinical laboratory. 2003;49(1–2):15–24. . 12593471

[31] Barak V, Goike H, Panaretakis KW, Einarsson R. Clinical utility of cytokeratins as tumor markers. Clinical biochemistry. 2004;37(7):529–40. 10.1016/j.clinbiochem.2004.05.009 . 15234234

[32] Oda M, Makita M, Iwaya K, Akiyama F, Kohno N, Tsuchiya B, et al High levels of DJ-1 protein in nipple fluid of patients with breast cancer. Cancer science. 2012;103(6):1172–6. 10.1111/j.1349-7006.2012.02267.x . 22404125 PMC7685089

[33] Zhao YS, Pang D, Wang F, Xue YW, Gao DN, Li H, et al Nipple aspirate fluid collection, related factors and relationship between carcinoembryonic antigen in nipple aspirate fluid and breast diseases in women in Harbin, PRC. Cancer epidemiology, biomarkers & prevention: a publication of the American Association for Cancer Research, cosponsored by the American Society of Preventive Oncology. 2009;18(3):732–8. 10.1158/1055-9965.EPI-08-0715 . 19273481

